# The Degradation Properties of MgO Whiskers/PLLA Composite In Vitro

**DOI:** 10.3390/ijms19092740

**Published:** 2018-09-13

**Authors:** Yun Zhao, Bei Liu, Hongwei Bi, Jinjun Yang, Wei Li, Hui Liang, Yue Liang, Zhibin Jia, Shuxin Shi, Minfang Chen

**Affiliations:** 1 School of Materials Science and Engineering, Tianjin University of Technology, Tianjin 300384, China; yun_zhaotju@163.com (Y.Z.); liubei8679@163.com (B.L.); earlybird.184@126.com (W.L.); huiliang2014@126.com (H.L.); 15102260655@163.com (Y.L.); zhibin225225@163.com (Z.J.); 13752048706@163.com (S.S.); 2 Key Laboratory of Display Materials and Photoelectric Device (Ministry of Education), Tianjin University of Technology, Tianjin 300384, China; 3 Tianjin Sannie Bioengineering Technology Co., Ltd., Tianjin 300384, China; bluewanli@126.com; 4 School of Environmental Science and Safety Engineering, Tianjin University of Technology, Tianjin 300384, China; tjyjj_2014@tjut.edu.cn

**Keywords:** biopolymers composites, MgO whiskers, PLLA, in vitro degradation

## Abstract

In this study, composite films of stearic acid–modified magnesium oxide whiskers (Sa–w-MgO)/poly-l-lactic acid (PLLA) were prepared through solution casting, and the in vitro degradation properties and cytocompatibility of the composites with different whisker contents were investigated. The results showed that the degradation behavior of the composite samples depended significantly on the whisker content, and the degradation rate increased with the addition of MgO content. Furthermore, the degradation of the composites with higher contents of whiskers was influenced more severely by the hydrophilicity and pH value, leading to more final weight loss, but the decomposition rate decreased gradually. Furthermore, the pH value of the phosphate buffer solution (PBS) was obviously regulated by the dissolution of MgO whiskers through neutralization of the acidic product of PLLA degradation. The cytocompatibility of the composites also increased remarkably, as determined from the cell viability results, and was higher than that of PLLA at the chosen whisker content. This was beneficial for the cell affinity of the material, as it notably led to an enhanced biocompatibility of the PLLA, in favor of promoting cell proliferation, which significantly improved its bioactivity, as well.

## 1. Introduction

Over the last few decades, poly(l-lactide) (PLLA) has been given significant attention, owing to its advantages of good biocompatibility and processability, biodegradability, and bioresorbability, which are desirable in the biomedical field, especially for bone repair [1,2,3,4]. However, some drawbacks limit its wider application [5,6,7,8,9,10], particularly its degradation properties, which are an extremely important factor in bone repair, as accumulation of the lactic acid degradation product from PLLA can cause issues such as aseptic inflammation in vivo, hydrophobicity and lower biological activity that are unfavorable to the viability of the bone cell, and biodegradation that cannot be well controlled according to the requirement of bone formation.

To solve these problems, many researchers have attempted to improve PLLA properties through various methods [11,12,13,14,15]. Generally, a composite prepared by adding inorganic fillers is very promising, as it has the potential to enhance the mechanical and thermal properties, to enhance the hydrophilicity and bioactivity, and to reduce aseptic inflammation, because the fillers used have good bioactivity and similar constituents to the inorganic compounds in bone, such as calcium phosphate. However, these fillers, such as hydroxyapatite (HA) [4,7,11,13], β-tricalcium phosphate (β-TCP) [5,6,15], and bioactive glass [16,17], are usually slightly soluble [18], which tends to limit their impact on neutralizing the acidic environment induced by PLLA hydrolysis.

Magnesium oxide (MgO), another inorganic filler with good biocompatibility, and bioactive capacity that is not toxic, has attracted much attention [19,20,21,22] for polymer modification. In addition to the aforementioned advantages, the Mg^2+^ released from MgO dissolving in water is beneficial to the protein synthesized via the activation of many enzymes, which contribute to the excellent bioactivity of MgO. It has been reported that the dissolution of MgO in vitro prominently influences the solution pH through alkaline degradation [19]. Recently, the researchers utilized the different shapes of MgO [9,19,20,21,22,23,24] as fillers and introduced them to PLLA for preparing composites. The results mostly showed that the mechanical properties and thermal behavior of PLLA improved significantly with the presence of MgO fillers, which was also proved in our works [24]. However, few reports have focused on the effect of MgO contents on the degradation behavior of the composite during the decomposition process. Ma et al. [19] obtained the n-MgO/PLLA composite by adding n-MgO modified by poly(ε-caprolactone) into a PLLA matrix, and the reduction of pH was suppressed to a certain extent, which was also demonstrated by us [22]. Luo et al. [9] studied the in vitro degradation properties of the MgO whiskers (w-MgO)/PLLA composite; their work mainly focused on the effect of whiskers on PLLA degradation for a long decomposition period. It is worth mentioning that because such composites have the potential for application in bone repair, it is crucial to investigate their degradability and the cytocompatibility performance of the initial degradation stage, especially in terms of how it is affected by the filler contents, which is greatly important for the materials used, as implant inescapably contacted with bone cell and tissue [18]. Meanwhile, it was also noted that the change in the hydrophilicity of the PLLA matrix improved with the addition of fillers such as organic montmorillonite (OMMT), which prominently accelerated the hydrolytic degradation of PLLA, playing another key role in controlling the degradation process of PLLA [25,26]. As for MgO, they greatly impacted not only the hydrolysis behavior of PLLA, but also its degradation conditions, by controlling the pH. Therefore, the complex process in which the hydrolysis of PLLA could be facilitated can be controlled by varying the whisker content as well as be retarded by changes in the pH value, simultaneously [19,27]. Furthermore, it was reported that the hydrolysis process of PLLA was also favorable for promoting the growth of an ordered crystalline structure, due to the easier movement of the chains and segment under the degrading state [28,29]. Additionally, studying the degradation behavior in vitro can provide fundamental experimental results for the degrading properties of PLLA used in bone repair in vivo.

In this work, the MgO whiskers were modified using stearic acid, and the chemical bondings were obtained through the interaction between them, as demonstrated by our previous work [24]. Because stearic acid is effective in modifying inorganic fillers like hydroxyapatite [30] and TiO_2_ [31] through changing the polarities, it was greatly helpful in improving the distribution of MgO whiskers and enhancing the interface of the PLLA composites. Then, this work undertook the detailed characterization of w-MgO/PLLA soaked in phosphate buffer solution (PBS) for different degrading periods and analyzed the influence of the whisker content on the composite degrading behaviors. The pH stability and cell affinity of the composite were also studied and evaluated by relative measurements and cell cultures to relate the changes in the composite degrading procedure to the material’s biocompatibility and bioactivity.

## 2. Results

### 2.1. XRD Measurement

**Figure 1:**
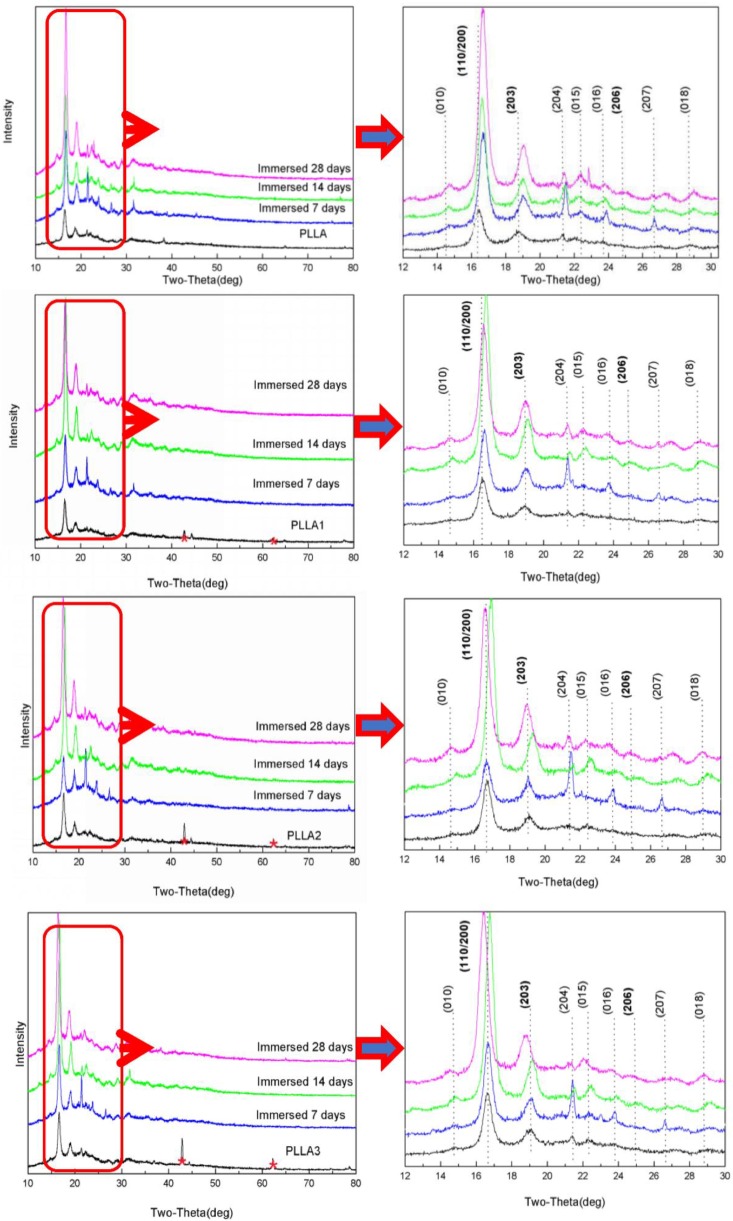
X-ray powder diffraction (XRD) patterns of the poly(l-lactide) (PLLA) and composite films at different degrading times and under partial enlargement.

In order to investigate the effect of MgO whiskers on the properties of PLLA, such as its crystalline structure and crystallization behavior, the samples of PLLA and composites were tested after being soaked using XRD and DSC, and the results of XRD testing are shown in Figure 1 and Table 1. Figure 1 shows that the intensity of diffraction characteristic peaks of PLLA, for example with 2θ values of 17.76° and 18.68°, corresponding to the (110/200) and (203) diffraction planes, respectively, clearly strengthened gradually, probably due to the surface decomposition and increment of PLLA crystallinity during degrading periods. Meanwhile, a slight shift of these peaks was noted with an increase in the immersion time, indicating the generation and transformation of different types of crystalline PLLA in the degrading process [32], especially between the forms of α’ and α. Specifically, the strength of 2θ at 24.82° for the (206) diffraction plane generated by the α’ form became more intense after 28 days’ immersion. Furthermore, these similar variations were also observed for the composite specimens, and the peak shifts were more prominent. This phenomenon can account for the accelerated degradation of the PLLA matrix, owing to the presence of MgO whiskers because of increased hydrophilicity [29]. Thus, a strong shift of the peaks was noted after 14 days. The little decrease in the 2θ value after 28 days of degradation seen for all composite samples, in comparison to that noted after 14 days, was attributed to the change in the relative content of the α’ and α forms caused by PLLA decomposition. Additionally, the characteristic peaks of MgO located at 43.5°, 62.5°, and 78.6°, corresponding to the (200), (220), and (222) diffraction planes, respectively, can also be found in the graphs of the composites, and PLLA3, with the highest content of 3 wt% MgO, showing the most significant peaks. However, the intensity of these peaks of MgO weakened gradually with progress in the experimental period: This change suggested a loss in the amount of MgO on the testing surface of composite films in the degrading process, showing no obvious peaks of the whiskers in the patterns of PLLA1, PLLA2, and PLLA3 for the 7 days in Figure 1.

### 2.2. DSC Analysis

**Figure 2:**
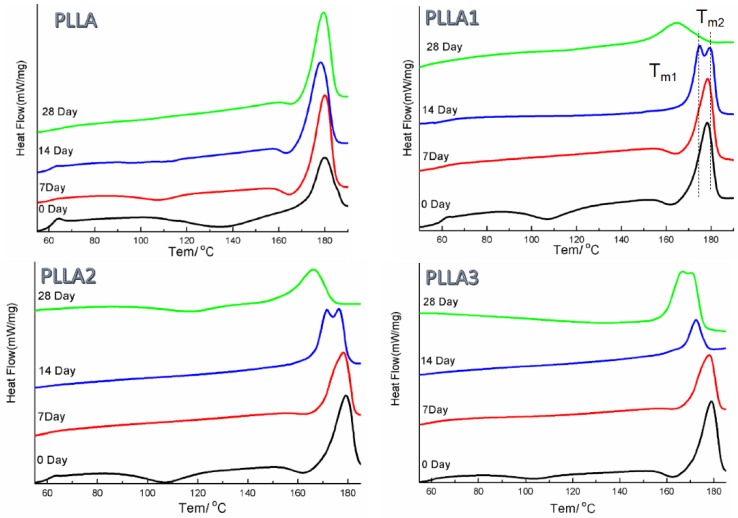
Secondary heating curves obtained by differential scanning calorimetry (DSC) for the samples after soaking in PBS for different degrading times.

The DSC data in the PLLA show the endothermic peaks of T_g_ and exothermic peaks of T_cc_ for the neat and composite materials for Day 0 in Figure 2. During the degradation process, the exothermic peaks observed around T_g_ and T_cc_ for the neat PLLA samples disappeared gradually, and meanwhile one weak exothermic peak was seen at the left side of the main endothermic peak, which was due to the α’→α transition [28] in the substrate from 7 days’ to 28 days’ immersion. Furthermore, it can be seen in Table 2 that the T_mc_ of the PLLA sample increased when it was immersed initially, and a small variation was observed in the value over the course of the experiment. This suggested an increased crystallinity of the specimen at the beginning of the procedure, caused by enhancement of the chain mobility, and particularly an increase in the amount of α form crystals, which was consistent with the XRD observation where a larger value of 2θ corresponding to the (110)/(200) crystal plane was obtained for PLLA after 28 days’ immersion. Compared to the control, the composites exhibited different crystalline behaviors. Because the whiskers effectively promoted PLLA bulk crystallization, there were obvious exothermic peaks of α’→α transitions observed for the composite samples on Day 0. Double fusion peaks and a prominent reduction in the final T_mc_ value were observed after 28 days, indicating that the composites decomposed faster than the control sample under the same degrading procedure. Moreover, the PLLA3 sample, after 28 days’ immersion, showed double fusion peaks similar to the ones shown by the PLLA1 and PLLA2 samples after 14 days, suggesting that an increased whisker amount was beneficial to inhibiting the degrading extent of the composites. This was also demonstrated by the T_mc_ value of PLLA1, 164.6 °C, which was the lowest among those exhibited by all specimens.

### 2.3. Surface and Fracture Morphologies of Sample Films after Degradation

**Figure 3:**
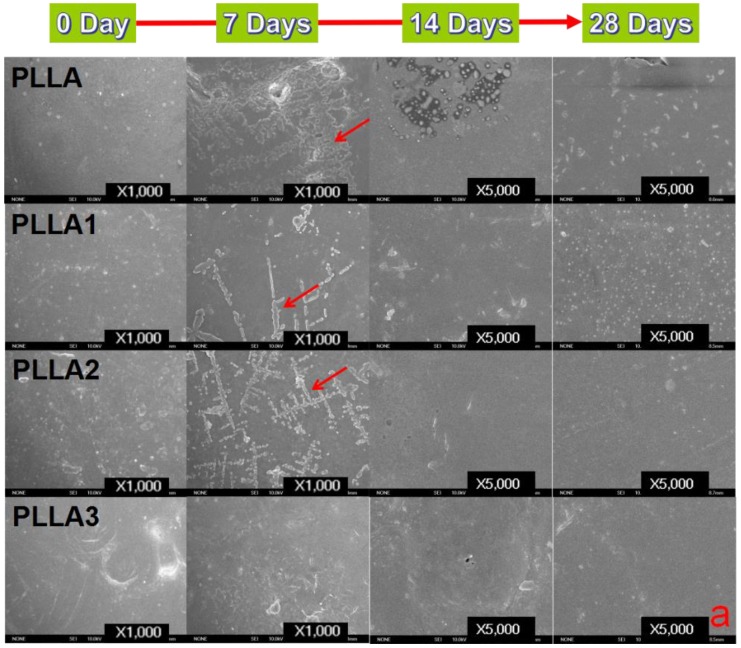

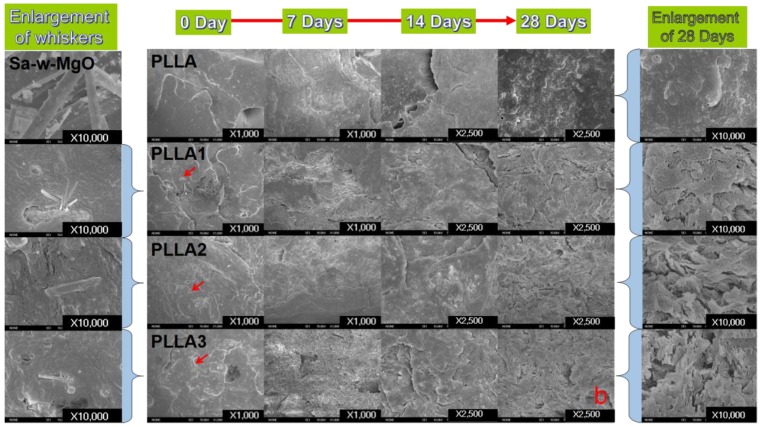
SEM micrographs of PLLA and composite films at different degradation times: (**a**) surface morphology (red arrows: crystalline region from the degradation); (**b**) fracture morphology (red arrows: MgO whiskers).

The surface and fracture morphologies (treated by liquid nitrogen for brittle fracture) of the sample films immersed in PBS for various degradation periods were obtained and are shown in Figure 3. It is obvious that the surfaces of all specimens degraded for 7 days became a little rougher in comparison to their initial smooth state, and it was also observed that the control film had a larger rough region than the composite ones, indicating an increase in the crystallinity of the composite on addition of MgO whiskers. This roughness observed after 7 days was probably due to the generation of the α’ form under the degradation, as shown in Figure 3a, of PLLA, PLLA1, and PLLA2, which had the bunch shape [33] because hydrolysis can facilitate matrix crystallinity through the movement of molecular chains [34,35] or reorganization and recrystallization in amorphous areas of the bulk [28]. The roughness was not obvious and not even observed after 14 days and 28 days, and a few small holes were found to be dispersed on the surface over the course of the sequential degrading process. This observation may be ascribed to the degradation of crystalline areas mentioned above with the progress of time [9], during which the rough surfaces disappeared.

To evaluate the effect of degradation on the inner structure of the films, the fracture morphology of the specimens after being treated for various degrading times are shown in Figure 3b. It shows that the samples had no obvious differences and still kept the dense microstructure for up to 28 days, and the fracture morphology of the composites still exhibited better toughness than neat PLLA. However, some micropores can be seen on the composites and can probably be attributed to the loss of the non-crystalline region during the PLLA hydrolysis degrading procedure, although more residual parts of the crystalline component were retained on them, which is consistent with Luo’s results [9].

### 2.4. The pH Values of the Immersing Solution, the Weight Loss and Mechanical Properties of Sample Films after Degradation

**Figure 4:**
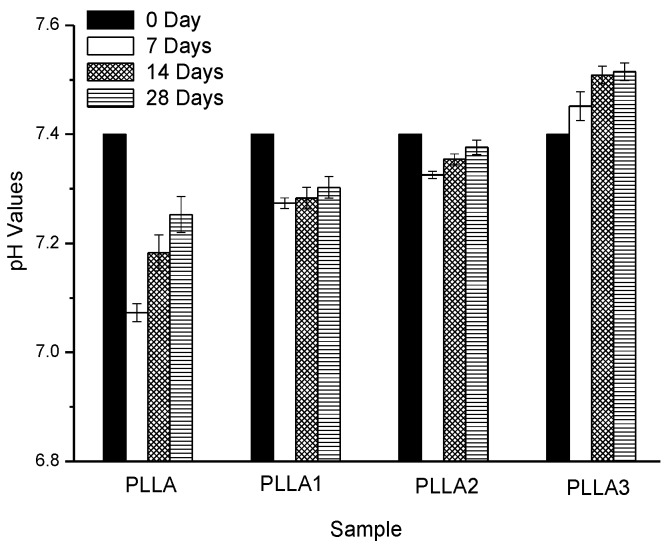
The pH value as a function of degradation time of the neat PLLA and composite films immersed in the phosphate buffer solution (PBS).

The pH values of the PBS after the degradation of neat PLLA and composite films are shown in Figure 4. It shows that the pH changes of the film samples in the testing period were not obvious, as pH varied in the range of 7.0 to 7.5. With an increase in the degradation time, the pH value of the soaking solution in the presence of neat PLLA film decreased from 7.4 (Day 0) to 7.25 (Day 28), and this was ascribed to the acidic product after the degradation of neat PLLA. Meanwhile, a slight buffering effect can be seen, attributed to the OH- released after dissolution of MgO in the PBS, owing to the added MgO whiskers in PLLA1. Obviously, increasing the incorporated content of whiskers in PLLA2 and PLLA3 resulted in an effective buffering on the pH value of the PBS, as the pH values observed were higher than those of the former. This suggested that the higher the amount of MgO whiskers added, the higher the pH value obtained would be and the more notable the buffering effect on the degrading solution, caused by the release of OH- because of MgO dissolving. However, it should also be noted that the higher value of pH, like alkalinity condition, was also able to accelerate PLLA degradation, and the degradation was even faster than its acidic autocatalytic degradation [36,37]. Generally, the degradation rate of PLLA in ambient pH value follows the order alkaline > acid > neutral. Therefore, it is illustrated that the much higher content of whiskers was not favorable for further enhancing the stability of the pH value of the PBS during the initial degrading stage of PLLA-based composite films.

**Figure 5:**
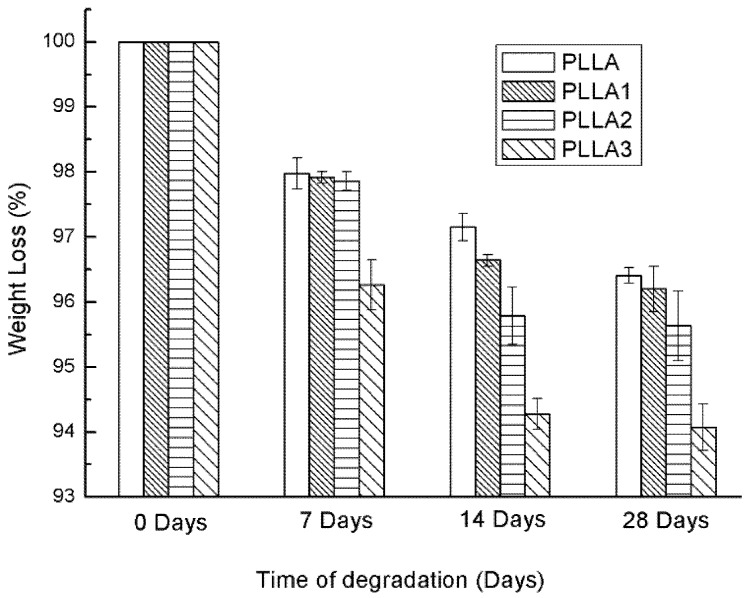
Weight losses as a function of degradation time of neat PLLA and composite films immersed in PBS.

For further analysis of the whiskers affecting the degradation of the PLLA bulk, the decomposition rate of the samples over time was investigated through their weight losses, and the results are shown in Figure 5. The weight of the sample films was reduced gradually during the degrading procedure. The weight losses of the composites PLLA2 and PLLA3 were comparatively more obvious, suggesting that a composite with a higher content of MgO whiskers exhibited a faster degradation rate, due to the increase in the pH value of the PBS, owing to MgO dissolution. Then, the weights of the films changed slightly after 28 days, because the non-crystalline region was degraded by the hydrolysis process and the residual part that was highly crystalline could not be hydrolyzed in the PBS, owing to the tight and regular arrangement of its molecular chains. Furthermore, PLLA1 degraded more slowly compared with PLLA in the later stage, which was consistent with the pH results and could be accounted for by the buffering effect of MgO neutralizing the acid from PLLA hydrolysis.

**Figure 6:**
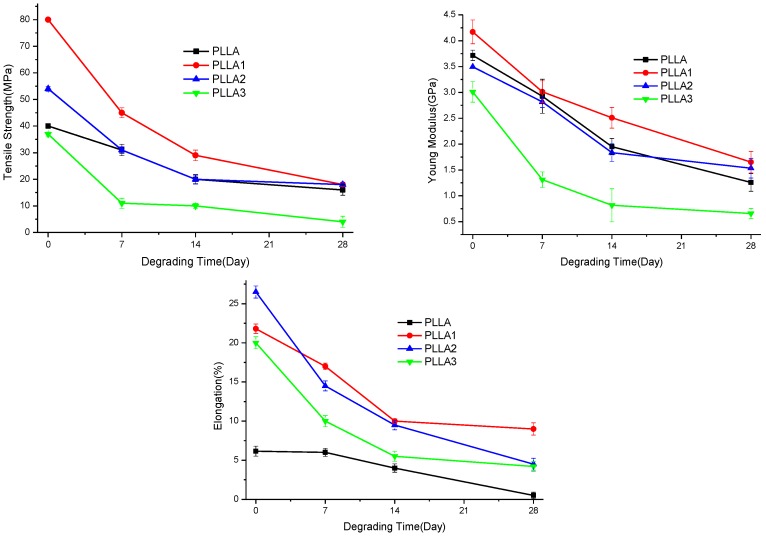
Mechanical properties of PLLA and composites at different degrading time.

The variation of mechanical properties of the samples during the degrading process is shown in Figure 6. Basically, the mechanical properties of PLLA and composite, including tensile strength, elastic modulus, and elongation at break, decreased as immersing time went. With increasing whisker content, the mechanical properties of the composite decreased notably; especially, the PLLA 3 with 3% whisker content exhibited a more obvious reduction in the tensile strength and Young modulus in comparison to pure PLLA. This deteriorated performance was probably due to the higher pH value of PLLA3 presented during the degradation. Meanwhile, it can be seen that the mechanical properties of composite were changed more greatly within the initially degrading stage, and this can probably be ascribed to the increasing hydrophilicity of composite induced by whiskers added [24].

### 2.5. Cell Experiments Results of Sample Films with Different Whisker Contents

**Figure 7:**
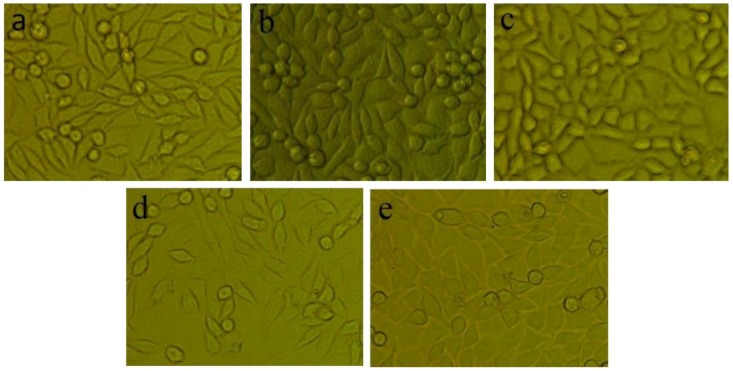
Morphology of the cell cultivated in leach liquor for 2 days (×100): (**a**) Negative control, (**b**) PLLA, (**c**) PLLA1, (**d**) PLLA2, (**e**) PLLA3.

**Figure 8:**
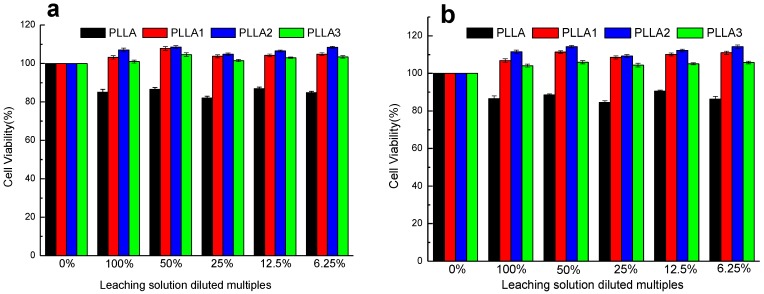
Cell viability of on PLLA, PLLA1, PLLA2, PLLA3 diluted 0, 2, 4, 8, and 16 times in leach liquor and culture for (**a**) 4 days and (**b**) 7 days, respectively.

The results of cell morphology observation and viability evaluations of the sample films are displayed in Figure 7 and Figure 8. It can be seen that small cells exhibited a good shuttle shape with an adherent and spreading morphology, whereas floating cells with spherical or round shapes observed in Figure 7 indicate a shape change and activity loss of the cells. Compared to that of the negative control group, the amount of cells showing loss in activity in PLLA was comparatively higher, but contrarily a decrease in the comparative number of floating cells was also observed in the composite samples in Figure 7c–e, with the cell shape being maintained similar to that of the negative control group. The variations of the cell states suggested that cells can show good growth and proliferation in the degrading solution of the composite films, because of the beneficial bioactivity and biocompatibility of MgO whiskers.

With the addition of MgO whiskers, cell viability in the composite films increased significantly compared to that of neat PLLA, as shown in Figure 8. Consistent with the cell morphology results shown in Figure 7, it exhibited a relatively higher cell viability on the composite than on neat PLLA, especially in the presence of PLLA2, with a 2 wt% content of whiskers. On extending the culture time, the composite had a higher cell number, indicating prominent cell proliferation. The improved cell viability in the presence of MgO whiskers was probably due to the buffering effect of MgO whiskers on the PBS and significant Mg^2+^ bioactivity as a cofactor in the cell metabolism acting on the cells. The former is helpful in providing a relatively stable pH state, and the latter is beneficial for enhancing cell growth rate.

## 3. Discussion

PLLA, as a widely used biodegradable polymer for biomedical applications, usually shows different degradation properties, depending on the degrading environmental conditions. In addition to the impact of enzymes in the body on its decomposition, the behavior of bulk hydrolysis mainly accounts for its degradation in vivo, and the performance of the implant in the early degrading process is extremely important, in particular, because new bone is usually intensively active during the early recovery stage [29,30,31,32].

**Figure 9:**
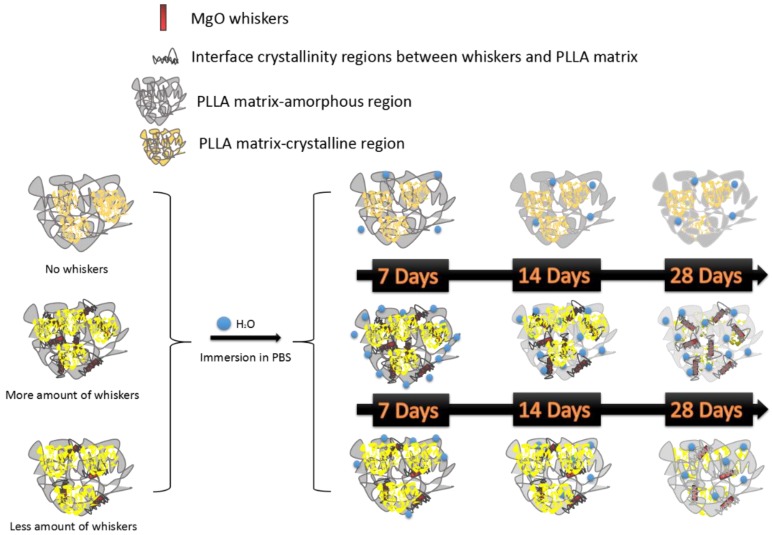
Diagram of in vitro degradation process for PLLA/magnesium oxide (MgO) whiskers composite films with different whisker contents.

Generally, the features of this hydrolysis include two sections with the decomposition of the non-crystalline region and that of the crystalline part, in that order [9]. The results of the XRD patterns and DSC curves for the pristine PLLA in Figure 1 and Figure 2 showed that the relative crystallinity of PLLA increased after degradation in PBS, as observed in Figure 3, demonstrating that the non-crystalline region in matrix was hydrolyzed first and, simultaneously, the crystalline structures of PLLA as α’-form were fabricated on inducing of hydrolysis through a chain or fragment movement in this process. On adding MgO whiskers, the crystallization degree, mechanical properties, and hydrophilicity of PLLA improved significantly, and the content of the whisker was undoubtedly also a key factor that affected the PLLA degrading properties [28,35]. Regarding the composite degradation behaviors, the initial change was similar to the degrading behaviors of the control PLLA, and the amorphous regions in the PLLA matrix were gradually destroyed, as shown by the corresponding changes in the patterns and curves of XRD and DSC. However, the hydrolysis rate of the PLLA bulk increased remarkably in the presence of whiskers, and the decomposing rate of the composites was prominently faster than that of the crystalline structure forming, which could be attributed to the hydrophilicity of the PLLA enhanced by the MgO whiskers. This hydrophilicity resulted in the notable acceleration and increment of water molecular seeping into the bulk (as shown in Figure 9), as reported previously [28]. As noted in Figure 1 and Figure 2, this increase in the degradation was limited in the specimen of PLLA3, which showed a lower rate of hydrolysis in comparison to PLLA2 in 28 days, and PLLA3 degradation showed a tendency of accelerating and then retarding, depending on the variation of T_m_, as shown in Table 2.

This was different from previously reported studies [25,28] mentioning an increase in the degradation rate with an increase in the amount of inorganic fillers, such as carbon nanotube–organic montmorillonite. This was probably due to two aspects: one was that the effect of the whiskers’ heterogeneous nucleation promoted the crystallization of PLLA, and an interface crystallinity [38] was also inclined to be introduced between the matrix and the whiskers, especially around the whisker regions, owing to the tight and regular packing of molecular chains. The numerous crystalline structures favorably prevented the water molecules permeating, in spite of the MgO whiskers increasing the hydrophilicity of the matrix, thereby inhibiting the water uptake of the materials. The other factor was the neutralizing effect of MgO whiskers that can effectively control the pH value of the PBS through their alkaline characteristics in solution. The MgO whiskers can impede the effect of acidic conditions and the autocatalysis caused by the PLLA hydrolysis, as a result of slowing down the degrading procedure of the PLLA. This hypothesis agrees with the SEM results of the surface and fracture morphologies of the samples, as shown in Figure 3. It should be noted that the influence of the dissolution of MgO on the pH of the PBS was more prominent for higher whisker contents. PLLA3 exhibited a higher degradation at the beginning of the immersion over 7 days, with a relatively higher weight loss, whereas the weight change was not obvious from 14 days to 28 days, which was similar to the mechanical properties in Figure 6. This was ascribed to the combined effects of the hydrophilicity and pH increase of the composites, which resulted in the fast loss of the amorphous region of the composites occurring initially. Then, the higher amount of OH- could control the pH value favorably, and this was beneficial in retarding the degrading course of the composites. Meanwhile, a small negative effect on cell viability of the PLLA3, as seen in Figure 7 and Figure 8, was also probably due to the pH increase of the sample at the earlier stage, and this can also be detrimental to the films’ biocompatibility and biological activity in terms of cell proliferation and viability. Simultaneously, the samples with 1 wt% and 2 wt% whisker contents were relatively more conductive for cell performance, and this was different from the results in previously reported studies, which selected a weight content of 5 wt% for the whiskers for investigating the effect of MgO whiskers on cell behaviors. Compared to the results above, the composites of our study showed faster degradation and beneficial pH regulation during the PLLA hydrolysis. The difference in the optimal whisker content in the studies performed was probably due to the type of surface modifiers of MgO whiskers. Therefore, the surface modifiers of whiskers can be an effective approach to controlling the degradation behaviors of the PLLA, and specific studies will be conducted on the modifier as a factor on composite material performance in following studies.

## 4. Materials and Methods

### 4.1. Materials

Powdered PLLA (*M_w_* = 417,000) was purchased from Daigang Biological Engineering Co., Ltd. (Jinan, China), and MgO whiskers were prepared in the laboratory according to the procedure described in the literature [24]. All other agents used were of analytical grade and did not require further treatment.

### 4.2. Preparation of MgO Whiskers and Their Modification

Using the procedure described in Reference [24], MgO whiskers were prepared according to a sequence of steps based on the chemical synthesis of a whisker precursor (magnesium carbonate hydrate) and its calcination. Na_2_CO_3_ (0.6 M) was added dropwise into an equal volume of MgCl_2_ (0.6 M) and stirred for 20 min. The mixture was aged at room temperature for 10 h and then filtered, washed, and dried at 80 °C for 3–4 h. The precursor was calcined at 750 °C for 4 h (the heating rate was 5 °C/min). The final product of this process was MgO whiskers (w-MgO).

Mixtures of 0.5 g of whiskers and 50 mL of ethanol were placed in three-necked flasks, which were subjected to ultrasonication in a bath to achieve full dispersion of the mixtures. The mixtures were then heated to 45 °C under reflux condensation. Stearic acid (Sa, 0.005 g) was dissolved into 20 mL of ethanol, added dropwise into each MgO suspension, and allowed to react for 1 h. The mixtures were then centrifuged, washed, and dried. The resultant product was denoted as Sa–w-MgO.

### 4.3. Preparation of Sa–w-MgO/PLLA Composite Films

PLLA/Sa–w-MgO composites with three different Sa–w-MgO contents (1, 2, and 3 wt%) were prepared through the solution casting method. The detailed procedure has been described in our previous reports [24]. Detailed information on the samples prepared is given in Table 3.

### 4.4. Degradation of the Composite Material in PBS

**Figure 10:**
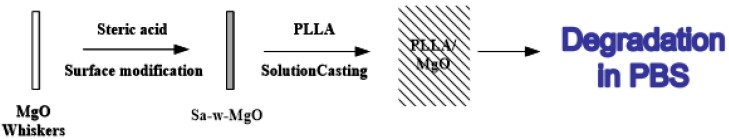
Schematic illustration of the preparation and degradation of the composite.

We carried out in vitro degradation of pure PLLA and composite samples of the dimensions 1 cm × 1 cm with a thickness of 1 mm by immersing them in 10 mL of a phosphate buffer solution in a rocking water bath at 37 °C for 7, 14, and 28 days. The specimens of each material were removed from PBS at the end of these periods. After rinsing with deionized water and removing the surface water using filter paper, the samples were weighed and then dried in vacuum at 40 °C to a constant weight. The weight losses were calculated using the following equation:

(1) $$W_{Loss}(\%)=\frac{m_{0}-m_{d}}{m_{0}}\times 100\%\;$$

where *W_Loss_* is the average degrading rate, and *m*_0_ and *m_d_* are the initial and final weights (after drying to constant weight), respectively. When the degradation was complete, the pH value of the PBS was determined by averaging the results of 3 independent measurements, obtained from three identical samples for each content. The process of film preparation and degradation is displayed in Figure 10.

### 4.5. Characterization

X-ray powder diffraction (XRD, Rigaku D/max/2500PC, Tokyo, Japan) was performed to obtain XRD patterns of the samples, using Cu Kα radiation with λ = 1.5418 Å, operating at 40 kV/100 mA, with a scanning speed of 8°/min. 

The morphology of the samples was characterized by field-emission scanning electron microscopy (FESEM, JOEL 6700F, Osaka, Japan, operating at 10 kV).

Differential scanning calorimetry (DSC) analyses were performed with a SETRAM SETSYS EVOLUTION16/18 (DSC 200F3, Netzsch Co., Selb, Germany). The samples, weighing approximately 5–8 mg each, were sealed in an aluminum pan, heated under nitrogen flow from room temperature to 220 °C at a heating rate of 20 °C/min, isothermally conditioned at 220 °C for 2 min, cooled to 0 °C, and then reheated to 220 °C at a heating rate of 10 °C/min.

**Figure 11:**
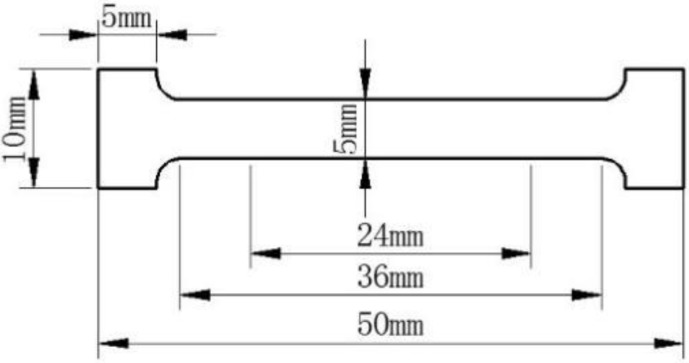
Detailed information of the samples used in mechanical testing.

The Figure 11 shows the detailed information of the samples’ shape used in mechanical testing. The measurements were taken in tension using a universal testing machine (Instron 5845, Boston, MA, USA) at an extension rate of 0.5 mm/min. There were three replicates of each sample tested, and the average and standard deviation of each set of replicates were calculated.

### 4.6. Cytotoxicity Testing

#### 4.6.1. Preparation of Material Extracts

With the ratio of film samples to culture medium set at 1 cm^2^/mL, the disinfected pristine PLLA and composite samples (1 cm × 1 cm) were immersed in an RPMI-1640 solution (Gibco, Grand Island, NY, USA) under a humidified atmosphere with 5% CO_2_ at 37 °C for 3 days to get the extraction medium. Prior to cell seeding, extracts were sterile-filtered through 0.2 mL syringe filters and then diluted by 2, 4, 8, and 16 times. The control groups involved the use of 10% fetal calf serum and 90% 1640 medium as negative controls.

#### 4.6.2. Culture of Murine Fibroblast Cells (L929)

Murine fibroblast cells (L929) were used to evaluate the in vitro cytotoxicity of the sample films. L-929 cells (Dingguo, Tianjin, China) were cultured in RPMI-1640 medium, supplemented with 10% fetal bovine serum (FBS) (Hyclone, Logan, UT, USA), 100 U/mL penicillin, and 100 U/mL streptomycin in a humidified incubator at 95% relative humidity and 5% CO_2_ at 37 °C. Then, the cells were cultured through the enzyme (Tryosin-EDTA, Sorabio, Beijing, China) digesting method.

#### 4.6.3. Cell Behavior and Viability

(1) After well sterilized, the pure PLLA and composite films were fixed in the 24-well flat-bottomed cell culture plates. Then, 10^4^ cells/500 μL medium were added in each well with the plates incubated for 2 days at 37 °C/5% CO_2_, and cell morphology was consequently observed by optical microscopy (Nikon ECLIPSE Ti inverted microscope, Tokyo, Japan).

(2) We incubated 24-well flat-bottomed cell culture plates with 10^4^ cells/500 μL medium in each well for 24 h to allow attachment. After the growth states were confirmed, the medium in the experimental group was removed and 500 μL extracts with various concentrations (dilutions of 0, 2, 4, 8, and 16 times) were placed in cell culture plates. The medium in the negative control group was replaced with 500 μL RPMI-1640 and 10% fetal calf serum, and at least 3 samples from each group were tested to confirm reproducibility. After incubating the cells in a humidified atmosphere for 4 and 7 days at 37 °C/5% CO_2_, 500 μL of 3-(4,5-dimethyl-2-thiazolyl)-2,5-diphenyl-2-*H*-tetrazolium bromide (MTT) (sigma, Burlington, MA, USA) (5 mg/mL) was added to each well and the samples were incubated with MTT in the incubator at 37 °C for 3 h, and 350 μL DMSO (Sorabio, China) was added into each well. Finally, the absorbance was recorded by a multimode detector on a microplate reader (Epoch, BioTek, Winooski, VT, USA) at a wavelength of 570 nm, and the OD value of each group was statistically analyzed to calculate cell viability. The cell viability were calculated using the following equation:

(2) $$\mathrm{Cell}\;\mathrm{viability}(\%)=\frac{{\mathrm{OD}}_{\mathrm{Experiment}}}{{\mathrm{OD}}_{\mathrm{Negative}\;\mathrm{control}}}\times 100\%\;$$

where OD_Experiment_ is the OD value from the sample films and OD_Negative control_ is the OD value from the negative control group, respectively.

## 5. Conclusions

In this study, the composites of PLLA/MgO whiskers with different whisker contents were soaked in PBS for relatively short treating periods to investigate their initial in vitro degrading properties while used as implants in the context of bone repair. It was demonstrated that an increase in the whisker content can accelerate the degradation rate of the PLLA matrix. The degrading procedure of composites with a higher content of whiskers was influenced more significantly by the hydrophilicity and pH value. As the results showed, the degradation of the composite began with loss of the non-crystalline region prior to destruction of the crystalline part, and the content of MgO whiskers present was a significant factor that influenced the degrading procedure. The cell morphology observation and cell viability measurements suggested that, in the presence of MgO whiskers, the composite showed higher cell viability and led to cells with flattened and narrow rhombus shapes, which enhanced the bioactivity of the PLLA matrix significantly. This PLLA/MgO biocomposite can be potentially utilized for bone repair with better performance.

## Figures and Tables

**Table 1:** The 2θ values of samples on Day 0.

Sample	Diffraction Plane								
	(010)	(110/200)	(203)	(204)	(015)	(016)	(206)	(207)	(018)
	2θ(°)								
PLLA	14.69	17.76	18.68	21.36	22.45	23.78	24.82	26.62	28.71
PLLA1	14.63	16.48	18.96	21.36	22.28	23.72	24.87	26.60	28.78
PLLA2	14.63	16.67	19.02	21.39	22.43	23.83	24.09	26.64	28.93
PLLA3	14.74	16.67	19.06	21.43	22.34	23.79	24.94	26.64	28.82

**Table 2:** Melting points of the samples used in DSC measurements.

Degradation Time	T_m_/°C			
	PLLA	PLLA1	PLLA2	PLLA3
0	179.8	178.2	179.2	179.1
7	180.0	178.5	178.1	177.9
14	178.6	174.9/179.4	171.4/176.4	172.4
28	179.6	164.6	166.3	166.8/170.4

**Table 3:** Detailed information of the samples.

Abbreviation	Sample	Whisker Content (*w*/*w*)
PLLA	PLLA	0
PLLA1	1 wt% Sa–w-MgO/PLLA	1/100
PLLA2	2 wt% Sa–w-MgO/PLLA	2/100
PLLA3	3 wt% Sa–w-MgO/PLLA	3/100

## References

[1] Alsberg E., Kong H.J., Hirano Y., Smith M.K., Albeiruti A., Mooney D.J. (2003). Regulating bone formation via controlled scaffold degradation. J. Dent. Res..

[2] Kakinoki S., Uchida S., Ehashi T., Murakami A., Yamaoka T. (2011). Surface Modification of Poly(l-lactic acid) Nanofiber with Oligo(d-lactic acid) Bioactive-Peptide Conjugates for Peripheral Nerve Regeneration. Polymers.

[3] Rogina A., Pribolšan L., Hanžek A., Gόmez-Estrada L., Ferrer G.G., Marijanović I., Ivanković M., Ivanković H. (2016). Macroporous poly(lactic acid) construct supporting the osteoinductive porous chitosan-based hydrogel for bone tissue engineering. Polymer.

[4] Wang X.J., Song G.J., Lou T. (2010). Fabrication and characterization of nano composite scaffold of poly(l-lactic acid)/hydroxyapatite. J. Mater. Sci..

[5] Yang Y.F., Zhao Y.H., Tang G.W., Li H., Yuan X.Y., Fan Y.B. (2008). In vitro degradation of porous poly(l-lactide-co-glycolide)/β-tricalcium phosphate (PLGA/β-TCP) scaffolds under dynamic and static conditions. Polym. Degrad. Stab..

[6] Liao L., Chen L., Chen A.Z., Pu X.M., Kang Y.Q., Yao Y.D., Liao X.M., Huang Z.B., Yin G.F. (2007). Preparation and characteristics of novel poly-l-lactide/β-calcium metaphosphate fracture fixation composite rods. J. Mater. Res..

[7] Shikinami Y., Matsusue Y., Nakamura T. (2005). The complete process of bioresorption and bone replacement using devices made of forged composites of raw hydroxyapatite particles/poly l-lactide (F-u-HA/PLLA). Biomaterials.

[8] Li X., Chu C.L., Liu L., Liu X.K., Bai J., Guo C., Xue F., Lin P.H., Chu P.K. (2015). Biodegradable poly-lactic acid based-composite reinforced unidirectionally with high-strength magnesium alloy wires. Biomaterials.

[9] Wen W., Zou Z.P., Luo B.H., Zhou C.R. (2017). In vitro degradation and cytocompatibility of g-MgO whiskers/PLLA composites. J. Mater. Sci..

[10] Nampoothiri K.M., Nair N.R., John R.P. (2010). An overview of the recent developments in polylactide (PLA) research. Bioresour. Technol..

[11] Jiang L.Y., Xiong C.D., Jiang L.X., Xu L.J. (2014). Effect of hydroxyapatite with different morphology on the crystallization behavior, mechanical property and in vitro degradation of hydroxyapatite/poly(lactic-co-glycolic) composite. Compos. Sci. Technol..

[12] Cifuentes S.C., Gavilán R., Lieblich M., Benavente R., González-Carrasco J.L. (2016). In vitro degradation of biodegradable polylactic acid/magnesium composites: Relevance of Mg particle shape. Acta Biomater..

[13] Shalumon K.T., Sheu C., Fong Y.T., Liao H.T., Chen J.P. (2016). Microsphere-Based Hierarchically Juxtapositioned Biphasic Scaffolds Prepared from Poly(Lactic-co-Glycolic Acid) and Nanohydroxyapatite for Osteochondral Tissue Engineering. Polymers.

[14] Kothapalli C.R., Shaw M.T., Wei M. (2005). Biodegradable HA-PLA 3-D porous scaffolds: Effect of nano-sized filler content on scaffold properties. Acta Biomater..

[15] Loher S., Reboul V., Brunner T.J., Simonet M., Dora C., Neuenschwander P., Stark W.J. (2006). Improved degradation and bioactivity of amorphous aerosol derived tricalcium phosphate nanoparticles in poly(lactide-co-glycolide). Nanotechnology.

[16] Yun H., Kim S., Park E.K. (2016). Bioactive glass-poly(ε-caprolactone) composite scaffolds with 3 dimensionally hierarchical pore networks. Mater. Sci. Eng. C.

[17] Fernandes J.S., Gentile P., Martins M., Neves N.M., Miller C., Crawford A., Pires R.A., Hatton P., Reis R.L. (2016). Reinforcement of poly-l-lactic acid electrospun membranes with strontium borosilicate bioactive glasses for bone tissue engineering. Acta Biomater..

[18] Johnson A.J.W., Herschler B.A. (2011). A review of the mechanical behavior of CaP and CaP/polymer composites for applications in bone replacement and repair. Acta Biomater..

[19] Ma F.Q., Lu X.L., Wang Z.M., Sun Z.J., Zhang F.F., Zheng Y.F. (2011). Nanocomposites of poly(l-lactide) and surface modified magnesia nanoparticles: Fabrication, mechanical property and biodegradability. J. Phys. Chem. Solids.

[20] Kum C.H., Cho Y., Seo S.H., Joung Y.K., Ahn D.J., Han D.K. (2014). A poly(lactide) stereocomplex structure with modified magnesium oxide and its effects in enhancing the mechanical properties and suppressing inflammation. Small.

[21] Kum C.H., Cho Y., Joung Y.K., Choi J., Park K., Seo S.H., Park Y.S., Ahn D.J., Han D.K. (2013). Biodegradable poly(l-lactide) composites by oligolactide-grafted magnesium hydroxide for mechanical reinforcement and reduced inflammation. J. Mater. Chem. B.

[22] Yang J.J., Cao X.X., Zhao Y., Wang L., Liu B., Jia J.P., Liang H., Chen M.F. (2017). Enhanced pH stability, cell viability and reduced degradation rate of poly(l-lactide)-based composite in vitro: Effect of modified magnesium oxide nanoparticles. J. Biomater. Sci. Polym. Ed..

[23] Wen W., Luo B., Qin X., Li C., Liu M., Ding S., Zhou C. (2015). Strengthening and toughening of poly(l-lactide) composites by surface modified MgO whiskers. Appl. Surf. Sci..

[24] Zhao Y., Liu B., You C., Chen M.F. (2016). Effects of MgO whiskers on mechanical properties and crystallization behavior of PLLA/MgO composites. Mater. Des..

[25] Chen H.M., Chen J.W., Chen J., Yang J.H., Huang T., Zhang N., Wan Y. (2012). Effect of organic montmorillonite on cold crystallization and hydrolytic degradation of poly(l-lactide). Polym. Degrad. Stab..

[26] Paul M.A., Delcourt C., Alexandre M., Degée P., Monteverde F., Dubois P. (2005). Polylactide/montmorillonite nanocomposites: Study of the hydrolytic degradation. Polym.Degrad. Stab..

[27] Fukushima K., Tabuani D., Dottori M., Armentano I., Kenny J.M., Gamino G. (2011). Effect of temperature and nanoparticle type on hydrolytic degradation of poly(lactic acid) nanocomposites. Polym. Degrad. Stab..

[28] Chen H.M., Feng C.X., Zhang W.B., Yang J.H., Huang T., Zhang N., Wang Y. (2013). Hydrolytic degradation behavior of poly(l-lactide)/carbon nanotubes nanocomposites. Polym. Degrad. Stab..

[29] Bose S., Tarafder S. (2012). Calcium phosphate ceramic systems in growth factor and drug delivery for bone tissue engineering: A review. Acta Biomater..

[30] Li Y.B., Weng W.J. (2008). Surface modification of hydroxyapatite by stearic acid: Characterization and in vitro behaviors. J. Mater. Sci. Mater. Med..

[31] Zhang L., Chen L., Wan H.Q., Chen J.M., Zhou H.D. (2011). Synthesis and Tribological Properties of Stearic Acid-Modified Anatase (TiO_2_) Nanoparticles. Tribol. Lett..

[32] Barry M., Pearce H., Cross L., Tatullo M., Gaharwar A.K. (2016). Advances in Nanotechnology for the Treatment of Osteoporosis. Curr. Osteoporos. Rep..

[33] Hutmacher D.W. (2000). Scafolds in tissue engineering bone and cartilage. Biomaterials.

[34] Rezwan K., Chen Q.Z., Blaker J.J., Boccaccini A.R. (2006). Biodegradable and bioactive porous polymer/inorganic composite scaffolds for bone tissue engineering. Biomaterials.

[35] Chen H.M., Shen Y., Yang J.H., Huang T., Zhang N., Wang Y., Zhou Z.W. (2013). Molecular ordering and α’-form formation of poly(l-lactide) during the hydrolytic degradation. Polymer.

[36] Tsuji H., Nakahara K. (2002). Poly(l-lactide). IX. Hydrolysis in Acid Media. J. Appl. Polym. Sci..

[37] Li S.M. (1999). Hydrolytic degradation characteristics of aliphatic polyesters derived from lactic and glycolic acids. J. Biomed. Mater. Res..

[38] Ning N.Y., Fu S.R., Zhang W., Chen F., Wang K., Deng H., Zhang Q., Fu Q. (2012). Realizing the enhancement of interfacial interaction in semicrystalline polymer/filler composites via interfacial crystallization. Prog. Polym. Sci..

